# Feasibility, acceptability and usability of InControl web-platform intervention for older adults with chronic musculoskeletal pain: A mixed-methods pilot study protocol

**DOI:** 10.1371/journal.pone.0360321

**Published:** 2026-10-14

**Authors:** David Oliveira, Nelson P. Rocha, Anabela G. Silva

**Affiliations:** 1 School of Health Sciences, University of Aveiro, Aveiro, Portugal; 2 Department of Medical Sciences, Institute of Electronics and Informatics Engineering of Aveiro (IEETA), University of Aveiro, Aveiro, Portugal; 3 RISE-HEALTH, School of Health Sciences, University of Aveiro, Aveiro, Portugal; PLOS: Public Library of Science, UNITED STATES OF AMERICA

## Abstract

**Background:**

Chronic musculoskeletal pain is highly prevalent among older adults and is associated with disability, reduced physical activity, and decreased quality of life. Digital health interventions can improve access to care and support pain self-management. InControl is a user-centered web-based platform that combines exercise and pain science education. The aim of this pilot study is to assess the usability and acceptability of the InControl platform and the feasibility of the research methods. This will help inform methodological improvements in the design of a future randomized controlled trial.

**Methods:**

A randomized, parallel-group, mixed-methods pilot study will be conducted with two groups: usual care group and the InControl intervention. Thirty-four participants aged 60 years or older with chronic musculoskeletal pain will be recruited at the Local Health Unit of Aveiro Region. The InControl intervention will last 8 weeks and will combine in-person sessions with remote interventions. Feasibility will be assessed using indicators of recruitment, retention, adherence, refusal, and dropout rates. Usability will be measured using the System Usability Scale, while acceptability and appropriateness will be assessed through a questionnaire and semi-structured interviews. Secondary outcomes will include pain, physical function, psychological factors, quality of life, and cognitive function. Data will be analyzed using descriptive statistics and qualitative thematic analysis.

**Discussion:**

The findings will inform the design of a future large-scale randomized controlled trial and contribute to the development of more effective digital interventions tailored to the needs of older adults with chronic musculoskeletal pain.

**Trial registration:**

This study is registered on clinicaltrials.gov, NCT07774416 (https://clinicaltrials.gov/study/NCT07774416?tab=study), August 18, 2026.

## Introduction

Increased life expectancy is a current trend worldwide [1]. The population aged 65 and older is expected to grow from 761 million in 2021 to 1.6 billion in 2050 [1]. A similar trend is observed in Portugal, reflecting a substantial increase in the aging index [2]. Chronic pain affects more than 50% of older adults, and it is also showing a tendency to increase over time [3]. Chronic musculoskeletal pain is associated with increased disability [4], reduced physical activity [5], higher rates of depression [3], and sleep problems [6]. It also negatively affects social and psychological well-being [4] and quality of life [7].

Pain represents one of the major challenges in healthcare, being one of the main reasons for seeking medical attention and contributing to substantial healthcare costs [4,8]. The prevalence of chronic conditions and multimorbidity in this population intensifies the burden on healthcare systems, challenging their capacity to respond on time [4]. To address these challenges, digital health interventions show potential to expand access to high-quality pain treatment [9], and to mitigate barriers such as distance and mobility limitations, which often restrict older adults’ access to in-person care [9,10].

The management of chronic musculoskeletal pain is demanding due to its multifactorial nature [6]. Exercise and pain science education are internationally recommended interventions for the management of chronic pain [11–15]. Existing studies [14,16,17] have demonstrated that exercise improves pain and physical function. On the other hand, pain science education changes beliefs related to chronic pain, promotes self-management and self-efficacy [18] and increases adherence to exercise [19]. However, evidence regarding the effectiveness and implementation of digital interventions for chronic musculoskeletal pain in older adults is conflicting [9]. A recent systematic review and meta-analysis found small, low to very low certainty effects on pain intensity and disability [9]. Moreover, many studies focus on only a single condition or a specific site of pain [9], although most older adults experience pain in more than one location [20], which limits the generalizability of the results to older adults with chronic musculoskeletal pain. Furthermore, research focuses primarily on effectiveness [9] rather than feasibility, acceptability, and usability.

The intervention contents within the InControl web-based platform were co-created with healthcare professionals and older adults who have lived experience of chronic musculoskeletal pain. InControl integrates exercise and pain science education tailored to this population. The platform’s goal is to deliver a technology-mediated intervention that helps reduce pain and mitigate its negative impact on older adults with chronic musculoskeletal pain. Because digital literacy, engagement, and adherence may be challenging in this population, a pilot study is required before evaluation in a large-scale randomized controlled trial. In this pilot study, the acceptability and usability, as well as the feasibility of the research procedures, will be evaluated in a real-world setting.

The pilot study aims to: i) assess the usability and acceptability of the InControl platform among older adults with chronic musculoskeletal pain; ii) determine the feasibility of using the proposed technological solution (InControl) over an 8-week period in a real-world setting, and iii) inform the methodological design of a randomized controlled trial to evaluate the cost-effectiveness of InControl.

## Methods

### Study design

This is a randomized, two-arm, parallel-group pilot study using mixed methods, including both quantitative and qualitative approaches. It includes feasibility, acceptability and usability components, conducted within an exploratory framework that follows the Standard Protocol Items Recommendations for the International Trials guidelines [21] (S1 File). This study received approval from the Ethics Committee of the Egas Moniz Health Alliance Clinical Academic Center, No. 21-CE-ICVS/CAC-EMHA/08.06.2026.

### Participants

#### Inclusion criteria.

To be eligible the participants must: i) be 60 years of age or older; ii) have chronic musculoskeletal pain, defined as pain that persists for more than three months and is located in the muscles, bones, joints, or tendons [22] at least once a week; iii) be able to use a computer and/or a tablet; iv) have a score of 3 or higher on the Numerical Pain Scale for average pain intensity over the past week; and v) show no cognitive impairment, as determined by the Montreal Cognitive Assessment (MoCA) (validated and with normative data for the Portuguese population) [23,24] and be available to participate in the study. The MoCA cutoff scores for the absence of cognitive impairment to be considered are adjusted for the participants’ age and educational level [primary school (1–4 years), middle school (5–9 years), high school (10–12 years), and college (>12 years)], with 1.5 standard deviations [24]: i) Ages 60–69: elementary school – 17 points, middle school – 21 points, high school and college – 22 points; ii) Ages 70–79: elementary school – 15 points, middle school – 18 points, high school – 20 points, and college – 22 points; iii) Over 80 years old: elementary school – 12 points, middle school – 22 points, high school – 17 points, and college – 19 points.

#### Exclusion criteria.

Participants will be excluded if they meet any of the following criteria: i) inability to read; ii) pain associated with serious conditions (e.g., tumors, fractures); iii) any acute, unstable, or decompensated medical conditions that prevent safe physical exercise. Examples include cardiovascular diseases (recent acute myocardial infarction, unstable angina, uncontrolled cardiac arrhythmias, severe symptomatic aortic stenosis, symptomatic or unstable heart failure, acute myocarditis or pericarditis, acute aortic dissection, active endocarditis, uncontrolled hypertension), respiratory conditions (acute pulmonary embolism, pulmonary infarction, chronic obstructive pulmonary disease, uncontrolled asthma, or recent respiratory exacerbations), metabolic conditions (uncontrolled diabetes mellitus), neurological conditions (stroke, traumatic brain injury, Parkinson’s disease), or acute systemic infection.

In addition, participants will be screened according to the American College of Sports Medicine’s Preparticipation Health Screening algorithm [25].

#### Recruitment and consent.

Participants for this study will be recruited through the primary healthcare centers of the Local Health Unit of Aveiro Region (ULSRA), which includes hospitals and primary healthcare centers, and from the community. Information will be disseminated to health professionals at the ULSRA. In addition, posters will be displayed in strategic locations, such as health centers, community settings, and institutions that organize activities for older adults, as well as through digital and print media. The promotional materials will provide detailed information about the study and eligibility criteria and will instruct individuals to contact the research team to express their willingness to participate.

All participants will receive verbal and written information about the study and will be required to provide written informed consent prior to participation.

#### Sample size.

Since this is a pilot study (S2 and S3 Files), the sample size was not determined statistically [26]. A total of 34 participants will be recruited and divided into two groups (n = 17 per group: intervention and control). This sample size accounts for an anticipated attrition rate of 25%, allowing for an estimated 12 participants per group to complete the study.

#### Randomization and group assignment.

The intervention will be delivered by members of the research team and physiotherapists from primary healthcare centers of the ULSRA.

Randomization will be performed at a cluster level, with the physical therapist serving as the unit of allocation. Physical therapists will be randomly assigned in a 1:1 ratio to either the usual care or the InControl intervention arms. This approach aims to minimize contamination between groups caused by the physical therapist and the exchange of information among participants from different groups. An independent researcher, not involved in participant recruitment or assessment, will generate a computer-generated sequence (simple randomization). Allocation will be concealed by this researcher until after the baseline assessment of the first participant is completed. Assessments will be conducted by a blinded assessor trained by the principal investigator. After randomization, all participants assigned to that physical therapist will receive the intervention designated for that cluster. Due to the nature of the intervention (exercise and education), it is not possible to blind participants and physical therapists to the intervention. The assessor will be blinded to the allocation of physical therapists and participants to the group throughout the trial. Unblinding of the outcome assessor is not anticipated, however, in the event of a severe adverse event requiring safety review, unblinding will be documented and reported. Since this is a feasibility study, six clusters are estimated, with each physical therapist managing between six and seven participants.

### Intervention

Each participant will be assigned to one of two groups (usual care or the InControl intervention).

#### Usual care.

This refers to the treatment that participants would normally receive in routine care (e.g., medication, physical therapy, or any other form of treatment). At the end of the treatment period, participants will report all treatments received during the intervention period.

#### InControl intervention.

Following the initial assessment at Week 0, participants will be introduced to the InControl platform with the assistance of a researcher to ensure familiarity and deliver technical support.

The intervention will last 8 weeks and will combine 4 in-person group sessions (in Weeks 1, 3, 5, and 7) with 20 remote sessions delivered via the InControl platform. Remote sessions will be conducted two or three times per week, at a time to be determined by the participant and on non-consecutive days. Adverse events occurring during the intervention period (e.g., falls) will be systematically recorded. The study provides insurance coverage for accidents that occur during or as a result of the intervention. Minor adverse events related to exercise will be recorded and managed by the physical therapists.

The in-person sessions will focus on platform support, documentation and resolution of technical or practical issues, reinforcement of pain science education content, adaptation and progression of exercises, assessment of functional goals and monitoring of adverse events. These sessions are designed to support adherence and enhance participants’ confidence in performing the intervention. Sessions will last approximately 60 minutes and will be conducted either at the School of Health Sciences of the University of Aveiro or at other facilities within the ULSRA, depending on the physical therapist responsible for delivery. The remote sessions include three main components: (i) completion of a functional goal-related task, (ii) pain science education and (iii) physical exercise. Each week, participants will complete the visualization of educational content and corresponding quizzes for about 20 minutes and exercise for approximately 30 minutes, on alternate days.

The functional goal is to support self-management strategies and improve or restore an individually selected activity of daily living. The selected activity will be one that the participant considers important but is currently limited in performing due to pain. Functional goals will be carried out outside the InControl platform, through either i) pacing—a self-management strategy in which participants learn to balance time spent active with time spent resting — or ii) graded exposure to movement/activity [27]. For example, a participant may set as their functional goal regaining the ability to prepare a full meal while standing. Initially, the participant can alternate five minutes of activity with five minutes of rest (pacing) or gradually increase the standing time during meal preparation with progressive increases over time.

Pain science education aims to improve participants’ understanding of pain as a biopsychosocial experience rather than a direct indicator of tissue damage, enabling pain reconceptualization, facilitating fear reduction and increase adherence to exercise [28,29]. Educational content includes topics such as the nature of chronic pain, the role of the nervous system and its sensitization, factors that modulate pain (e.g., emotions, sleep), factors that contribute to the maintenance of pain, the distinction between pain and injury, and pain self-management strategies. Content will be delivered through standardized videos, audio, and text developed in collaboration with clinicians and older adults with chronic pain. Participants will view two videos per week during the first four weeks, with optional review thereafter. Knowledge acquisition is reinforced through quizzes following each video.

Each participant will receive a tailored exercise program adapted to their abilities over the course of 8 weeks. This program will be repeated for two weeks and may be modified during in-person sessions or upon the participant’s request when clinically appropriate. Participants may contact the physical therapist for guidance, share difficulties or to report symptom changes. Exercise prescription will be personalized, involving both progression and regression in the difficulty/level of the exercises, as well as changes in exercise modalities (mobility, balance, strengthening, or stretching), in order to ensure the participant’s safety, well-being, and symptom tolerance. Exercise intensity should be light (3–4) to moderate (5–6), as measured by the Modified Borg Scale, which is used to assess subjective perception of exertion [25,30]. Each exercise session will last between 30 and 40 minutes and will include a warm-up (5 minutes), main exercise component (15–30 minutes), and cool-down (5 minutes). Each session must include mobility and strengthening exercises and may incorporate other components as needed.

### Assessment instruments and procedures

#### Primary outcomes.

To assess feasibility, the following indicators will be calculated:

i) Inclusion rate: the ratio between the number of participants enrolled in the study and the total number of individuals contacted;
ii) Refusal rate: ratio between the number of individuals who refused to participate in the study and the number of individuals contacted;
iii) Exclusion rate: ratio between the number of individuals excluded due to not meeting the inclusion criteria and the total number of individuals contacted;
iv) Dropout rate: ratio between the number of participants who discontinue the study and the number of participants who completed the baseline assessment;
v) Retention rate: ratio between the number of participants who completed the final assessment relative to those assessed at baseline.

Usability will be assessed at the end of the 8-week intervention using the validated Portuguese version of the System Usability Scale [31] referring to the InControl platform.

Acceptability of both study procedures and intervention will be assessed through a structured questionnaire (S4 and S5 Files) and a semi-structured interview (S6 and S7 Files). The questionnaire will include 10 statements to be rated on a 5-point Likert scale (1 = “strongly disagree” and 5 = “strongly agree”). The semi-structured interview will be conducted individually in a quiet setting using a predefined interview guide. The guide includes open-ended questions addressing participants’ experiences of the study procedures and intervention, including information received before the study, waiting time, perceived benefits and difficulties, barriers to adherence in daily life, platform usability, suggestions for improvement, and overall satisfaction with the program. All interviews will be audio-recorded and securely stored in an encrypted server at the University of Aveiro. Recordings will be retained for up to 2 weeks before transcription, after which they will be deleted.

Adherence to the intervention is defined as the completion of at least 75% of the scheduled sessions [32,33]. Attendance at in-person sessions will be recorded, and platform usage data (frequency and duration of use) will be automatically collected. In case of two consecutive missed sessions, the research team will contact participants by telephone to identify barriers and deliver support.

InControl usage data will be automatically recorded, including the number and duration of platform uses. The time required to administer the assessment battery will be recorded to evaluate its appropriateness. Finally, predefined criteria (Table 1) will determine whether the study protocol is ready to proceed to a full randomized controlled trial, requires amendments or is not feasible without a major protocol restructuring. These thresholds are based on previous studies [34,35].

**Table 1:** Progression criteria to proceed to the randomized controlled trial.

Criterion	Proceed	Proceed with protocol amendments	Do not proceed without major restructuring
**Related to procedures**			
**Inclusion rate** (elegibility)	At least 1 in 4 eligible individuals are included (25% or higher)	At least 1 in 6 eligible individuals are included (15–24%)	At least 1 in 10 eligible people are included (less than 10%)
**Refusal rate** (recruitment)	30% or less Most of those contacted agree to participate	Between 31% and 50% Moderate refusal rate – review contact protocol	Over 50% Most participants decline – review criteria or strategy
**Exclusion rate**	50% or less Appropriate eligibility criteria	Between 51% and 70% Review exclusion criteria	Over 70% Criteria are too restrictive
**Dropout rate**	Less than 25% of participants drop out of the intervention	Between 25% and 40% of participants drop out of the intervention	More than 40% of participants drop out of the intervention
**Retention rate**	75% or more of participants complete the initial and final assessments	Between 60% and 74% of participants complete the initial and final assessments	Less than 60% of participants complete the initial and final assessments
**Intervention-related**			
**Intervention adherence**	75% or more of the participants completed at least three-quarters of the InControl intervention sessions	At least 65% of participants completed at least three-quarters of the InControl intervention sessions	Less than 65% of participants completed at least three-quarters of the InControl intervention sessions
**Usability** (SUS)	The average score on the SUS scale is 80 points or higher	The average SUS score ranged from 68 to 79 points	The average SUS score was below 68 points and the required changes are not feasible within an acceptable timeframe
**Acceptability** (questionnaire)	75% or more of the participants agree or strongly agree with the acceptability questions	At least 50% of participants agreed or strongly agreed with the acceptability questions	Less than 50% of participants agreed or strongly agreed with the acceptability questions
**Adverse events** (number of adverse events/number of sessions)	Adverse events in 10% or fewer of participants, associated with or occurring during the intervention	Adverse events occurred in 20% or fewer of participants, either associated with or occurring during the intervention	Adverse events occurred in 21% or more of participants, either associated with or occurring during the intervention

#### Secondary outcomes.

The Initiative on Methods, Measurement, and Pain Assessment in Clinical Trials (IMMPACT) [36] recommends that trials on pain assess the following six core domains: i) pain, ii) physical function, iii) emotional function, iv) perception of improvement, v) adverse symptoms and events, and vi) detailed information about the participants. All secondary outcomes described below will be considered to assess their appropriateness for inclusion in the future study.

Participants will be evaluated for the following variables:

i) pain severity and interference [Brief Pain Inventory (BPI) – severity and interference subscales], location and number of sites [body chart], pain phenotype [PainDETECT (PDQ)];
ii) performance [handgrip strength, walking speed test, 5 sit-to-stand tests], physical activity level [Rapid Assessment of Physical Activity (RAPA)];
iii) catastrophizing [Pain Catastrophizing Scale (PCS)], kinesiophobia [Tampa Scale of Kinesiophobia (TSK)], depressive symptoms [Geriatric Depression Scale (GDS)];
iv) Global Impression of Change [Patient Global Impression of Change (PGIC)] – only assessed at the post-intervention stage;
v) risk of sarcopenia [Strength, assistance with walking, rising from a chair, climbing stairs, and falls (SARC-F)];
vi) sociodemographic and clinical data, and quality of life [EQ-5D-5L e EQ-VAS].

Evidence regarding the association between pain and cognitive impairment in older adults is inconsistent [37,38]. Therefore, cognitive function [Montreal Cognitive Assessment] will also be assessed both at baseline and following intervention. The Portuguese versions of these instruments are valid and reliable.

The platform will store the number and duration of sessions, exercise prescriptions, and whether or not the exercises were completed. Given that the intervention will be delivered by multiple physical therapists, intervention fidelity will be assessed. It is defined as the extent to which an intervention is implemented as specified in the study protocol [32,39]. Two independent researchers will evaluate interventions recorded on the InControl platform, which will be selected randomly. The evaluation will be based on a structured questionnaire, with responses on a 1-to-5 Likert scale (1 = “not at all confident” and 5 = “completely confident”) regarding adherence to protocol. The study methods are outlined in Fig 1.

**Fig 1:**
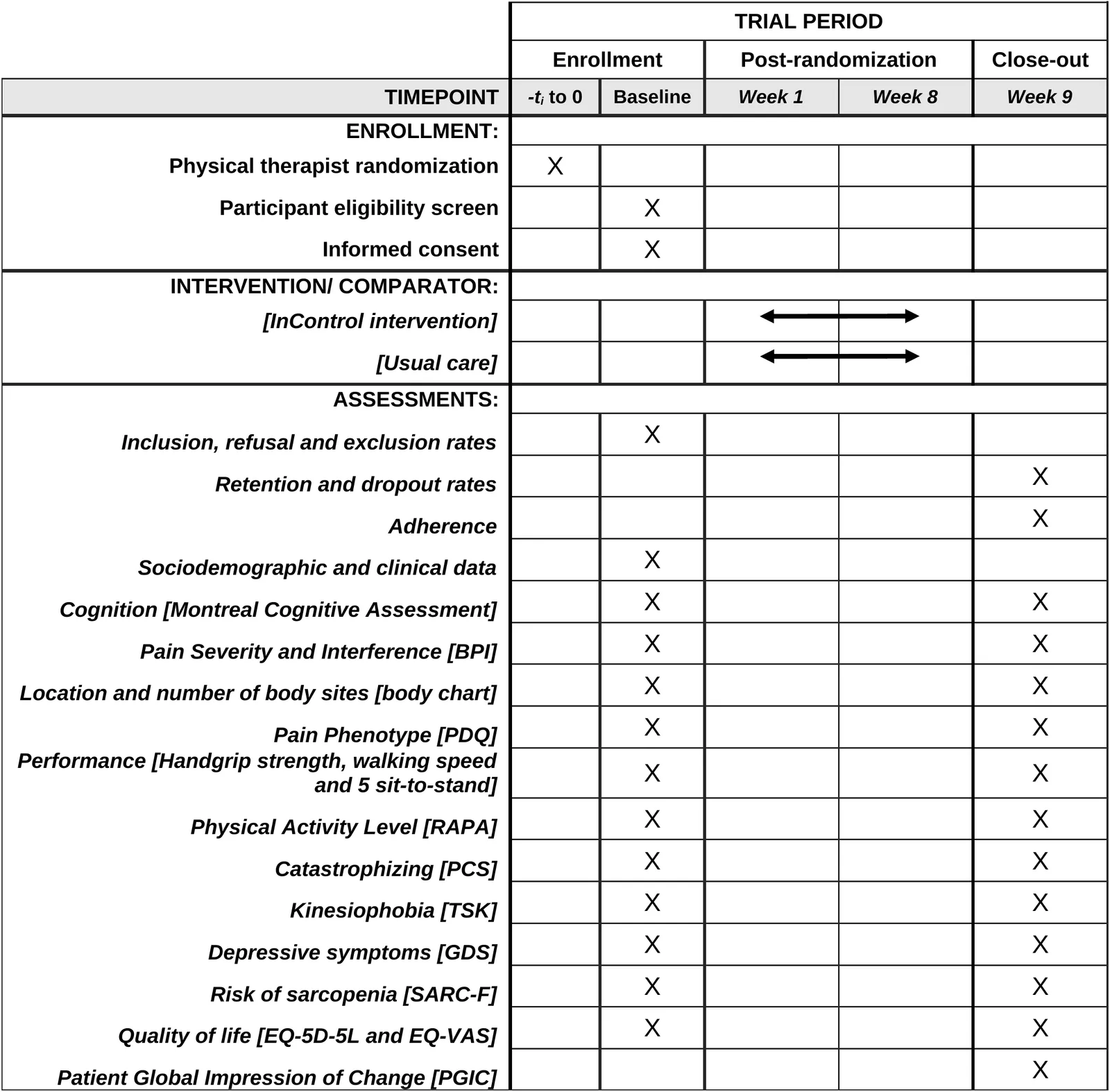
Schedule of enrollment, interventions, and assessments of participants.

#### Authentication and data management of InControl.

Access to the InControl platform will be via a computer or tablet with internet access. Participants who do not have access will be provided with a tablet with internet access for the duration of the intervention. Each participant will receive individual login credentials and instructions for secure platform use.

The principal investigator will generate login credentials for the physical therapists who will then create accounts for their allocated participants only.

All data collected through InControl will relate only to system usage and will be stored on a secure server that complies with current regulations, with regular backups will be performed. The key linking participant identities to codes will be accessible only to two research team members and store in a password-protected file.

Given the pilot and low-risk nature of the trial, no independent data monitoring committee has been established, and no interim analysis are planned. Instead, the research team will continuously manage, verify, and supervise overall data quality, trial safety, and protocol compliance, with the principal investigator holding final authority to modify or suspend the study if needed.

De-identified individual participant data, data dictionary, statistical code, and study materials will be available from the corresponding author upon reasonable request following the publication of the main results.

### Data analysis plan

Quantitative data will be analyzed using IBM Statistical Package for Social Sciences (SPSS) 31.0.0. Descriptive statistics, means, and standard deviations for continuous variables, and frequencies and percentages for ordinal/nominal variables, will be used to characterize the sample, the results related to feasibility, and the results regarding secondary variables. Missing data will be documented and reported. Due to the small sample size and exploratory nature of this pilot trial, subgroup and sensitivity analyses will not be performed.

Qualitative analysis of the data will be conducted using thematic analysis, following three steps: reading, coding, and thematization [40]. The interviews will initially be transcribed in full and read independently by three researchers, who will produce summaries of the main concepts and compare them with one another [40]. Next, the data will be coded by assigning units of meaning (phrases), and categories with a shared meaning will then be grouped into themes [40]. In addition, quantitative indicators will be provided demonstrating the number of participants who shared the summary of the idea under the same theme.

### Timeline

Participant recruitment is scheduled to begin in September 2026 and is expected to be completed by November 2026. As the intervention lasts 8 weeks, data collection is anticipated to conclude by January 2027. Following data collection, the results will be anonymized, aggregated, and analyzed to prepare and submit a manuscript for scientific publication after that date.

## Discussion

Digital health interventions have been growing in popularity by expanding access to quality health care. Therefore, it is necessary to implement InControl in real-world settings to assess its usability and acceptability over the course of an 8-week intervention. Assessing feasibility will identify methodological challenges and optimize the design of the future randomized controlled trial.

### Strengths and limitations

This protocol of a pilot study demonstrates transparency in the research process and presents several strengths. The InControl platform is user-centered, with its pain science education and exercise components co-created by older adults and physical therapists, enhancing its relevance and appropriateness. The intervention will consist of a blended format, combining in-person sessions with remote sessions delivered via InControl, as requested by the co-creators of the content. The cluster randomization design facilitates minimizing the risk of contamination between groups. On the other hand, the mixed-methods design provides complementary quantitative and qualitative evidence regarding intervention implementation.

However, some limitations must be acknowledged. Due to the nature of the intervention, either the participants or the physical therapists cannot be blinded, which may introduce some bias. Additionally, the small sample size may limit the generalizability of some data. Finally, given that this is a digital intervention, variability in digital literacy among Portuguese older adults may affect engagement and adherence to the intervention, and because the eligibility criteria will exclude individuals who are unable to use a computer or tablet, we will be contributing to the digital divide

## Supporting information

S1 File SPIRIT 2025 checklist. (PDF)

S2 File Study protocol (Portuguese). (PDF)

S3 File Study protocol (English). (PDF)

S4 File Questionnaire (Portuguese). (PDF)

S5 File Questionnaire (English). (PDF)

S6 File Interview guide (Portuguese). (PDF)

S7 File Interview guide (English). (PDF)
