# Supplementary material for: Feasibility, acceptability and usability of InControl web-platform intervention for older adults with chronic musculoskeletal pain: A mixed-methods pilot study protocol
Source: PLoS One. 2026 Oct 14;21(10):e0360321. doi: 10.1371/journal.pone.0360321 (PMC13667814; doi:10.1371/journal.pone.0360321)
Supplement: Supplementary file 2 [file pone.0360321.s002.pdf]

Formulário para pedido de parecer sobre questões éticas no âmbito de projetos académicos, de desenvolvimento ou de investigação científica

1. CARATERIZAÇÃO DO PROJETO

1.1 Designação do estudo

Viabilidade, aceitabilidade e usabilidade da plataforma web *InControl* – estudo piloto de métodos mistos

1.2 Data de início e de conclusão

Início: julho 2026; Término: janeiro 2027

1.3 Enquadramento

Em Portugal, a população com mais de 65 anos deverá crescer de 2,6 para 3,1 milhões entre 2014 e 2100, acentuando o envelhecimento populacional[1]. A dor crónica musculoesquelética (DCM) afeta mais de 50% dos adultos mais velhos[2], contribuindo para maior incapacidade[3], menor qualidade de vida[4] e aumento dos custos em saúde[3,5]. Desta forma, intensifica a pressão sobre os sistemas de saúde, desafiando a sua capacidade de resposta[3]. As intervenções de saúde digitais surgem como uma alternativa promissora, com potencial para ampliar os cuidados de qualidade [6], ao reduzir as barreiras no acesso a cuidados presenciais, como a distância e as limitações da mobilidade[6,7]. No entanto, considerando a possibilidade de uma baixa literacia digital nas pessoas com 65 anos ou mais[6] é importante incluir participantes de uma faixa etária próxima, mas ligeiramente mais baixa. Para gestão desta condição multifatorial[8], o exercício e a educação em ciência da dor são intervenções recomendadas internacionalmente[9–12]. O exercício proporciona benefícios na redução da intensidade e melhoria da incapacidade associada[12–14] e a educação modifica crenças relacionadas com a DCM, promove a autogestão e a autoeficácia[15] e aumenta a adesão ao exercício[16].

Este estudo realiza-se no âmbito do projeto *InControl* - Desenvolvimento centrado no utilizador e avaliação do custo-efetividade de um programa digital de gestão da dor para pessoas mais velhas, COMPETE2030-FEDER-00781500.

1.4 Objetivos

O *InControl* é uma plataforma web, cocriada com profissionais de saúde e pessoas mais velhas com experiência vivida de DCM, que integra exercício e educação em ciência da dor. Esta visa reduzir a dor e o impacto negativo nesta população, através de mediação tecnológica. Antes de ser testada num estudo randomizado de larga escala, a plataforma *InControl* e os procedimentos de investigação serão avaliados num estudo piloto em contexto real. Assim, os objetivos do estudo são: i) avaliar a usabilidade e aceitabilidade da plataforma InControl por pessoas mais velhas com DCM, ii) determinar a viabilidade de usar a tecnologia proposta, ao longo de 8 semanas e iii) informar o desenho metodológico de um estudo randomizado controlado, para avaliar o custo-efetividade do *InControl*.

2. PROCEDIMENTOS

2.1 Desenho do estudo

Este é um estudo piloto randomizado e paralelo de métodos mistos (qualitativo e quantitativo), com componentes de viabilidade e usabilidade, seguindo as recomendações CONSORT[17] para estudos piloto.

2.2 PARTICIPANTES

2.2.1 Critérios de inclusão: i) ter 60 ou mais anos; ii) ter DCM definida como uma dor que persiste por mais de três meses, é sentida nos músculos, ossos, articulações ou tendões[18], pelo menos uma vez por semana; iii) saber usar um computador e/ou um tablet; iv) ter pontuação igual ou superior a 3 na Escala Numérica da Dor para a intensidade da dor média na última semana e iv) sem défice cognitivo, com base no teste da função cognitiva *Montreal Cognitive Assessment*, ajustados à idade e nível de escolaridade e v) ter disponibilidade para participar. O preenchimento do

formulário de pré-participação (Apêndice – Formulário Pré-participação) será acompanhado e verificado pela equipa de investigação.

2.2.2 Critérios de exclusão: i) não saber ler; ii) dor associada a causas graves (ex. tumores, fraturas), iii) quaisquer condições médicas agudas, instáveis ou descompensadas que impeçam a realização de exercício físico de forma segura. Adicionalmente, os participantes serão triados segundo o algoritmo de *Preparticipation Health Screening do American College of Sports Medicine*[19] (Apêndice – Formulário Pré Participação).

2.3 Recrutamento

Os participantes serão recrutados na Unidade Local de Saúde da Região de Aveiro e na comunidade. O estudo será apresentado junto dos Médicos de Medicina Geral e Familiar e dos Fisioterapeutas da ULS de Aveiro para que o possam, também eles, divulgar. Serão, ainda, afixados cartazes (nos centros de saúde, na comunidade e em instituições que dinamizem atividades para pessoas mais velhas) e em publicações digitais ou em jornais. A divulgação terá informações detalhadas sobre o estudo e contatos da equipa de investigação para que os interessados em participar manifestem a sua disponibilidade, junto da equipa de investigação. Todos os participantes receberão informações verbais e escritas e terão de assinar um consentimento livre e esclarecido (Apêndice – Consentimento informado).

2.4 Tamanho da amostra

Tratando-se de um estudo piloto, o tamanho da amostra não foi determinado estatisticamente[17]. Serão recrutados 34 participantes, distribuídos por dois grupos (n=17 controlo, n=17 intervenção). Considerou-se uma taxa de perda de 25%, o que permitirá terminar o estudo com 12 participantes em cada grupo.

2.5 Randomização e alocação ao grupo

A randomização será efetuada pela Investigadora Principal do projeto (Anabela G. Silva; que não participa no recrutamento ou tratamento das pessoas com dor), realizada por cluster, sendo o fisioterapeuta que administra a intervenção a unidade de alocação. Este método visa a minimização da contaminação do fisioterapeuta aos grupos e a troca de informações entre participantes de diferentes grupos. Os fisioterapeutas que administrarão a intervenção serão alocados aleatoriamente num rácio 1:1 a um dos braços, o da intervenção habitual ou da intervenção *InControl*, através de uma sequência gerada por computador. A ocultação da randomização será mantida por um investigador independente, até que se complete a avaliação do primeiro participante. As avaliações serão realizadas por um avaliador cego. Uma vez atribuídos a um grupo, todos os participantes atribuídos a esse fisioterapeuta receberão a intervenção estipulada para esse cluster. Devido ao tipo de intervenção (exercício e educação), é impossível o cegamento dos participantes e fisioterapeutas à intervenção. O avaliador será cego em relação à alocação dos fisioterapeutas e participantes ao grupo. Estimam-se 6 clusters (cada cluster terá um fisioterapeuta que tratará entre 6 e 7 participantes).

3. INTERVENÇÃO

Cada participante será alocado aleatoriamente a um de dois grupos (intervenção habitual ou intervenção *InControl*).

3.1 Intervenção habitual

Corresponde à intervenção que o participante receberia habitualmente (pode incluir medicação, fisioterapia ou qualquer outra). Será pedido ao participante no final do período alocado à intervenção -que indique a intervenção que recebeu (apêndice – instrumentos).

3.2 Intervenção *InControl*

Após a avaliação inicial, na semana 0, os participantes explorarão a plataforma *InControl* com o apoio de um investigador para familiarização e suporte técnico com a mesma. Durante 8 semanas, os participantes realizarão 4 sessões presenciais em grupo (nas semanas 1, 3, 5 e 7) e 20 sessões à distância via plataforma *InControl*. As sessões à distância terão uma frequência de 2 ou 3 vezes por semana, consoante a concretização da sessão presencial, em dias alternados e em horário a definir pelo participante.

3.2.1 Sessões presenciais

Um total de 4 sessões presenciais, distribuídas por 8 semanas de forma quinzenal. Com durações de 60 minutos, decorrerão na Escola Superior de Saúde da Universidade de Aveiro ou noutros locais da ULS da Região de Aveiro. Estas visam o suporte técnico da plataforma o reforço da educação em ciência da dor e o ajuste personalizado da componente de exercício. Servirão, ainda, para monitorizar possíveis eventos adversos. O estudo dispõe de um seguro de responsabilidade civil.

3.2.2 Sessões à distância

Um total de 20 sessões à distância, distribuídas por 8 semanas (2 ou 3 sessões em cada semana). Em cada semana, a pessoa tem que: i) realizar a tarefa definida no âmbito do objetivo funcional; ii) ver os vídeos de educação e responder aos respetivos quizzes (cerca de 20 minutos); e ii) realizar 3 sessões de exercício em dias intercalados (cerca de 30 minutos cada sessão). O objetivo funcional focar-se-á na melhoria ou recuperação de uma atividade da vida diária considerada importante pelo participante, limitada pela sua dor. Esta componente será realizada fora da plataforma *InControl*, através de estratégias de autogestão como o: i) pacing, que ensina a equilibrar o tempo em atividade com o tempo em repouso – por exemplo, em vez de preparar a refeição toda de uma vez, trabalha cinco minutos e alterna com 5 minutos de descanso ou ii) exposição gradual ao movimento/atividade, onde o participante iniciará a atividade em pé durante dez minutos, aumentando progressivamente o tempo (por exemplo, mais dois minutos) consoante o aumento da tolerância. Em suma, o objetivo consiste em facilitar a autogestão em atividades significativas para a pessoa. Os conteúdos de educação abrangem a definição de dor crónica, o papel do sistema nervoso e da sua sensibilização, os fatores que modulam a dor (emoções, sono, etc.) e que contribuem para a sua manutenção, a distinção entre dor e lesão e estratégias de autogestão. Esta componente, desenvolvida numa fase anterior em cocriação com médicos, fisioterapeutas e pessoas mais velhas com dor, será administrada via vídeos, áudio e texto padronizados. No final dos vídeos, os participantes responderão aos respetivos quizzes (i.e., perguntas sobre os conteúdos). Cada participante terá um plano de exercícios individualizado e ajustado às suas capacidades, ao longo de 8 semanas, que será repetido durante duas semanas, revisto e alterado nas sessões presenciais. A intensidade do exercício deverá ser leve (3-4) a moderada (5 a 6), medida pela Escala de Borg Modificada, utilizada como medida de perceção subjetiva de esforço[19,20]. Cada sessão de exercício seguirá uma estrutura padronizada, com um total entre 30 e 40 minutos, ajustável à tolerância de cada participante e à fase da intervenção, devendo conter obrigatoriamente exercícios de mobilidade e de fortalecimento, podendo integrar as demais componentes. Esta estrutura deve ser composta pelo aquecimento (5 minutos), componente principal (15 a 30 minutos) e retorno à calma (5 minutos).

3.3 Avaliação da fidelidade da intervenção

A fidelidade da intervenção refere-se ao grau em que uma intervenção é aplicada conforme previsto no protocolo[21,22] e irá focar-se nas ações dos fisioterapeutas que entregam a intervenção com *InControl*. Dois investigadores avaliarão, independentemente, intervenções registadas na plataforma *InControl*, que serão selecionadas aleatoriamente. A

avaliação basear-se-á num questionário estruturado, com respostas numa escala de *Likert* de 1 a 5, onde 0 corresponde a “nada confiante” e 5 a “totalmente confiante” quanto à fidelidade ao protocolo.

3.4 Adesão dos participantes à intervenção

A adesão do grupo *InControl* à intervenção define-se como o número de participantes que completam a intervenção[21,23], como critério de adesão à realização de, pelo menos, 75% das sessões. Será avaliada pelo registo de presenças nas sessões presenciais e pelos dados eletrónicos de acesso à plataforma (frequência e tempo). Caso o participante não realize duas sessões consecutivas, a equipa de investigação efetuará um contato telefónico para averiguar os motivos e oferecer o suporte necessário.

3.5 Administração da intervenção

A administração da intervenção será realizada por membros da equipa de investigação e por fisioterapeutas da ULS da Região de Aveiro.

4. INSTRUMENTOS E PROCEDIMENTOS DE AVALIAÇÃO

4.1 Variáveis principais

Para avaliar a viabilidade da intervenção, importa calcular as seguintes taxas: i) Taxa de inclusão: o rácio entre o número de participantes incluídos no estudo e o número total de pessoas contactadas; ii) Taxa de recusa: o rácio entre o número de indivíduos que se recusaram a participar no estudo e o número de indivíduos contactados; iii) Taxa de exclusão: o rácio entre o número de indivíduos excluídos por não cumprirem os critérios de inclusão e o número total de indivíduos contactados; iv) Taxa de abandono: o rácio entre o número de participantes que abandonaram o estudo e o número de participantes que completaram a avaliação inicial; v) Taxa de retenção: o rácio entre o número de participantes que completaram a avaliação final e o número de participantes que completaram a avaliação inicial.

Para avaliar a usabilidade, após a intervenção (8 semanas), será solicitado aos participantes que preencham a versão Portuguesa da *System Usability Scale* sobre a plataforma *InControl* (Apêndice – entrevistas).

A aceitabilidade e adequação dos procedimentos e do programa de intervenção será avaliada através de questionário estruturado e de uma entrevista semiestruturada para explorar a perceção dos participantes. O questionário, terá 10 afirmações para responder numa escala de *Likert* de 1 a 5, onde 1 corresponde a “discordo fortemente” e 5 a “concordo fortemente”. O guião da entrevista incluirá perguntas sobre a adequação do processo, a estrutura do programa, recursos utilizados, a experiência com a plataforma InControl e os aspetos relacionados com a inclusão do programa rotina diária. Apenas o áudio das entrevistas será gravado, transferido para um servidor da UA para uma pasta encriptada onde permanecerá por um período máximo de 2 semanas até à transcrição, altura em que será apagado (Apêndice – entrevistas).

Serão extraídos dados de utilização do *InControl*, registados automaticamente como o número de utilizações e duração das mesmas. O tempo de aplicação da bateria de avaliações será estimado para aferir a sua adequabilidade.

4.2 Variáveis secundárias

A *Initiative on Methods, Measurement, and Pain Assessment in Clínical Trials* (IMMPACT)[24] recomenda a avaliação de 6 domínios em estudos sobre dor: i) dor - severidade e interferência da dor, localização e número de locais, fenótipo; ii) função física – desempenho físico (força de preensão manual, Teste de velocidade da marcha, 5 *sit-to-stand Test*], nível de atividade física; iii) função emocional - catastrofização, cinesiofobia e sintomas depressivos; iv) perceção de melhoria

– perceção global de mudança (avaliada no pós intervenção); v) sintomas e eventos adversos – risco de sarcopenia, quedas, vi) informação detalhada sobre os participantes – dados sociodemográficos e clínicos e qualidade de vida. As evidências de uma associação entre a dor e o compromisso cognitivo são contraditórias em pessoas mais velhas com DCM[25,26]. Assim torna-se importante a avaliação da função cognitiva tanto no momento inicial, como no pós-intervenção. As versões portuguesas dos instrumentos são válidas e fiáveis (Apêndice – Instrumentos).

5. AUTENTICAÇÃO E GESTÃO DE DADOS NO INCONTROL

O acesso à plataforma *InControl* será realizado através de um computador ou tablet, com acesso à internet. Aos participantes que não tenham acesso, será disponibilizado um tablet com internet durante o período da intervenção. Cada participante receberá uma chave de acesso para login na plataforma *InControl* e instruções de como usá-la de forma segura em sua casa. Numa primeira fase, serão atribuídas credenciais, que os participantes deverão alterar a palavra-passe no primeiro acesso.

A investigadora principal criará as credenciais de acesso dos fisioterapeutas que irão administrar a intervenção e cada um destes criará os utilizadores para os participantes que irá tratar (apenas e só para os seus).

Os dados recolhidos com o *InControl* dizem respeito apenas a dados de utilização do sistema e serão armazenados num servidor da Universidade de Aveiro. O servidor cumpre os regulamentos em vigor, serão feitas cópias de segurança regulares, e os dados são encriptados.

A autenticação dos participantes no sistema é feita através de um código pessoal e apenas os investigadores Anabela G. Silva e David Oliveira terão acesso à chave que faz a correspondência entre o participante e o respetivo código de identificação (pseudoanonimização). Este código será guardado no computador da investigadora Anabela Silva, que é propriedade da Universidade de Aveiro, em ficheiro Excel protegido por palavra-passe.

6. ANÁLISE DE DADOS

Os dados quantitativos serão analisados utilizando o *software IBM SPSS* 31.0.0. Para caraterizar a amostra, os resultados de viabilidade e variáveis secundárias, serão utilizadas estatísticas descritivas: médias e desvios padrão para variáveis contínuas e frequências e percentagens para variáveis ordinais/nominais.

A análise qualitativa seguirá uma análise temática em três etapas: leitura, codificação e tematização[27]. As entrevistas transcritas integralmente, serão analisadas independentemente por três investigadores, cujos resumos e conceitos serão posteriormente comparados[27]. Durante a codificação dos dados, serão atribuídas unidades de significado (frases) e agrupando categorias em temas comuns[27]. Adicionalmente, serão fornecidos indicadores quantitativos do número de participantes que partilharam as ideias identificadas em cada tema.

BIBLIOGRAFIA

1. Instituto Nacional de Estatística. Projeções de População Residente em Portugal. (2025). 2. Wettstein, M. & Tesarz, J. Increasing pain prevalence and intensity among middle-aged and older adults: Evidence from the German Ageing Survey. *Journal of Psychosomatic Research* **168**, 111233 (2023). 3. Domenichiello, A. F. & Ramsden, C. E. The silent epidemic of chronic pain in older adults. *Prog Neuropsychopharmacol Biol Psychiatry* **93**, 284–290 (2019). 4. Fong, H. P.-Y. *et al.* Determinants of health-related quality of life in older people with chronic musculoskeletal pain: a cross-sectional study. *BMC Geriatr* **24**, 119 (2024). 5. Cohen, S. P., Vase, L. & Hooten, W. M. Chronic pain: an update on burden, best practices, and new advances. *The Lancet* **397**, 2082–2097 (2021). 6. Silva, A. G., Santos, A. J., Andias, R. & Rocha, N. P. Effectiveness of digital pain management for older adults with musculoskeletal pain: systematic review with meta-analysis. *Front. Pain Res.* **6**, 1657014 (2025). 7. Valentijn, P. P. *et al.* Digital Health Interventions for Musculoskeletal Pain Conditions: Systematic Review and Meta-analysis of Randomized Controlled Trials. *J Med Internet Res* **24**, e37869 (2022). 8. Welsh, T. P., Yang, A. E. & Makris, U. E. Musculoskeletal Pain in Older Adults. *Med Clin North Am* **104**, 855–872 (2020). 9. Schofield, P. *et al.* Evidence-based clinical practice guidelines on the management of pain in older people – a summary report. *British Journal of Pain* **16**, 6–13 (2022). 10. Reid, M. C., Eccleston, C. & Pillemer, K. Management of chronic pain in older adults. *BMJ* https://doi.org/10.1136/bmj.h532 (2015) doi:10.1136/bmj.h532. 11. Horgas, A. L. Pain Management in Older Adults. *Nursing Clinics of North America* https://doi.org/10.1016/j.cnur.2017.08.001 (2017) doi:10.1016/j.cnur.2017.08.001. 12. Kechichian, A. *et al.* Multimodal Interventions Including Rehabilitation Exercise for Older Adults With Chronic

Musculoskeletal Pain: A Systematic Review and Meta-analyses of Randomized Controlled Trials. *Journal of Geriatric Physical Therapy* **45**, 34 (2022). 13. Silva, H. de J. *et al.* Group-based exercise reduces pain and disability and improves other outcomes in older people with chronic non-specific low back pain: the ESCAPE randomised trial. *Journal of Physiotherapy* **71**, 108–116 (2025). 14. Syroyid Syroyid, I., Cavero-Redondo, I. & Syroyid Syroyid, B. Effects of Resistance Training on Pain Control and Physical Function in Older Adults With Low Back Pain: A Systematic Review With Meta-analysis. *Journal of Geriatric Physical Therapy* **46**, E113 (2023). 15. Heleno, E., Andias, R. & Silva, A. G. What do community-dwelling older adults with chronic pain value in a program of combined pain neuroscience education plus exercise? *Patient Education and Counseling* **104**, 3072–3078 (2021). 16. Siddall, B. *et al.* Short-term impact of combining pain neuroscience education with exercise for chronic musculoskeletal pain: a systematic review and meta-analysis. *PAIN* **163**, e20 (2022). 17. Eldridge, S. M. *et al.* CONSORT 2010 statement: extension to randomised pilot and feasibility trials. *BMJ* i5239 (2016) doi:10.1136/bmj.i5239. 18. Perrot, S. *et al.* The IASP classification of chronic pain for ICD-11: chronic secondary musculoskeletal pain. *PAIN* **160**, 77 (2019). 19. Riebe, D. *et al.* Updating ACSM's Recommendations for Exercise Preparticipation Health Screening. *Medicine & Science in Sports & Exercise* **47**, 2473 (2015). 20. Baptista, F. M., Andias, R., Rocha, N. P. & Silva, A. G. A Practice Guide for Physical Therapists Prescribing Physical Exercise for Older Adults. *Journal of Aging and Physical Activity* **32**, 771–783 (2024). 21. Páez, A., Nunan, D., McCulloch, P. & Beard, D. The influence of intervention fidelity on treatment effect estimates in clinical trials of complex interventions: a metaepidemiological study. *Journal of Clinical Epidemiology* **177**, 111610 (2025). 22. Teresi, J. A., Yu, X., Stewart, A. L. & Hays, R. D. Guidelines for Designing and Evaluating Feasibility Pilot Studies. *Med Care* **60**, 95–103 (2022). 23. Chan, C. L., Taljaard, M., Lancaster, G. A., Brehaut, J. C. & Eldridge, S. M. Pilot and feasibility studies for pragmatic trials have unique considerations and areas of uncertainty. *J Clin Epidemiol* **138**, 102–114 (2021). 24. Dworkin, R. H. *et al.* Core outcome measures for chronic pain clinical trials: IMMPACT recommendations. *Pain* **113**, 9–19 (2005). 25. Yuan, H. *et al.* Contribution of pain to subsequent cognitive decline or dementia: A systematic review and meta-analysis of cohort studies. *International Journal of Nursing Studies* **138**, 104409 (2023). 26. de Aguiar, G. P. C. G. *et al.* Persistent pain and cognitive decline in older adults: a systematic review and meta-analysis from longitudinal studies. *PAIN* **161**, 2236 (2020). 27. Saunders, C. H. *et al.* Practical thematic analysis: a guide for multidisciplinary health services research teams engaging in qualitative analysis. *BMJ* **381**, e074256 (2023).
