# Supplementary material for: Feasibility, acceptability and usability of InControl web-platform intervention for older adults with chronic musculoskeletal pain: A mixed-methods pilot study protocol
Source: PLoS One. 2026 Oct 14;21(10):e0360321. doi: 10.1371/journal.pone.0360321 (PMC13667814; doi:10.1371/journal.pone.0360321)
Supplement: Supplementary file 3 [file pone.0360321.s003.pdf]

Form for requesting an opinion on ethical issues in academic, development, or scientific research projects

1. PROJECT CHARACTERIZATION

1.1 Title of the study

Feasibility, acceptability, and usability of the InControl web platform – a mixed-methods pilot study

1.2 Start and completion dates

Start: July 2026; End: January 2027

1.3 Background

In Portugal, the population aged 65 and older is expected to grow from 2.6 to 3.1 million between 2014 and 2100, accentuating population aging[1]. Chronic musculoskeletal pain (CMP) affects more than 50% of older adults[2], contributing to greater disability[3], lower quality of life[4], and increased healthcare costs[3,5]. Consequently, it intensifies pressure on healthcare systems, challenging their capacity to respond[3]. Digital health interventions emerge as a promising alternative, with the potential to scale quality care[6] by reducing barriers to in-person care, such as distance and mobility limitations[6,7]. However, considering the possibility of low digital literacy among people aged 65 or older[6], it is important to include participants from a close, yet slightly younger age group. For the management of this multifactorial condition[8], exercise and pain science education are internationally recommended interventions[9–12]. Exercise provides benefits in reducing pain intensity and improving associated disability[12–14], while education modifies beliefs related to CMP, promotes self-management and self-efficacy[15], and increases exercise adherence[16]. This study is conducted within the scope of the InControl project – User-centered development and cost-effectiveness evaluation of a digital pain management program for older adults, COMPETE2030-FEDER-00781500.

1.4 Aims

InControl is a web-based platform, co-created with healthcare professionals and older adults with lived experience of CMP, that integrates exercise and pain science education. It aims to reduce pain and its negative impact on this population through technological mediation. Before being tested in a large-scale randomized trial, the InControl platform and the research procedures will be evaluated in a real-world pilot study. Thus, the objectives of the study are: i) to evaluate the usability and acceptability of the InControl platform by older adults with CMP, ii) to determine the feasibility of using the proposed technology over an 8-week period, and iii) to inform the methodological design of a randomized controlled trial evaluating the cost-effectiveness of InControl.

2. PROCEDURES

2.1 Desenho do estudo

This is a mixed-methods (qualitative and quantitative) parallel randomized pilot study, with feasibility and usability components, following the CONSORT recommendations for pilot studies[17].

2.2 PARTICIPANTS

2.2.1 Inclusion criteria: i) aged 60 or older; ii) having CMP defined as pain that persists for more than three months, felt in muscles, bones, joints, or tendons, at least once a week; iii) knowing how to use a computer and/or a tablet; iv) having a score equal to or greater than 3 on the Numeric Rating Scale for average pain intensity over the past week; iv) no cognitive impairment, based on the Montreal Cognitive Assessment test, adjusted for age and education level; and v) availability to participate. The completion of the pre-participation form (Appendix – Pre-participation Form) will be monitored and verified by the research team.

2.2.2 Exclusion criteria: i) inability to read; ii) pain associated with serious causes (e.g., tumors, fractures); iii) any acute, unstable, or decompensated medical conditions that prevent the safe performance of physical exercise. Additionally, participants will be screened using the American College of Sports Medicine[19] Preparticipation Health Screening algorithm (Appendix – Pre-participation Form).

2.3 Recruitment

Participants will be recruited from the Local Health Unit of the Aveiro Region (ULS Aveiro) and the community. The study will be presented to General Practitioners and Physical Therapists at ULS Aveiro so they can also help promote it. Additionally, posters will be displayed (in health centers, the community, and institutions offering activities for older adults) alongside digital publications and newspaper listings. Promotional materials will include detailed information about the study and contact details for the research team, allowing interested individuals to express their willingness to participate directly to the team. All participants will receive verbal and written information and must sign an informed consent form (Appendix – Informed Consent).

2.4 Sample size

As this is a pilot study, the sample size was not statistically determined[17]. A total of 34 participants will be recruited and allocated into two groups (n=17 control, n=17 intervention). A drop-out rate of 25% was estimated, which will allow the study to conclude with 12 participants in each group.

2.5 Randomization and group allocation

Randomization will be conducted by the Principal Investigator of the project (Anabela G. Silva, who is not involved in recruitment or treatment delivery) using cluster randomization, with the physical therapist administering the intervention serving as the unit of allocation. This method aims to minimize therapist contamination across groups and prevent information sharing between participants in different groups. Physical therapists delivering the intervention will be randomly allocated in a 1:1 ratio to either the usual care or the InControl intervention arm using a computer-generated sequence. Allocation concealment will be maintained by an independent researcher until baseline evaluation of the first participant is complete. Assessments will be conducted by a blinded outcome assessor. Once allocated, all participants assigned to a given physical therapist will receive the intervention designated for that cluster. Due to the nature of the intervention (exercise and education), blinding of participants and physical therapists is not possible. The assessor will remain blinded to the group allocation of both physical therapists and participants. Six clusters are estimated (each cluster comprising one physical therapist treating between 6 and 7 participants).

3. INTERVENTION

Each participant will be randomly allocated to one of two groups: usual care or the InControl intervention.

3.1 Usual care

Usual care corresponds to the intervention the participant would typically receive, which may include medication, physical therapy, or any other treatment. At the end of the allocated intervention period, participants will be asked to report the intervention they received (Appendix – Instruments).

3.2 InControl intervention

Following the baseline assessment, in week 0, participants will explore the InControl platform with the support of a researcher to become familiar with the platform and receive technical assistance. Over 8 weeks, participants will attend 4 in-person group sessions (in weeks 1, 3, 5, and 7) and complete 20 remote sessions via the InControl platform. Remote

sessions will be delivered 2 or 3 times per week, depending on whether an in-person session is scheduled that week, on alternate days, and at a time chosen by the participant.

3.2.1 In-person sessions

A total of 4 in-person sessions will be delivered every two weeks over an 8-week period. Each session will last 60 minutes and will take place at the School of Health Sciences of the University of Aveiro or at other sites within the Local Health Unit of the Aveiro Region. These sessions aim to provide technical support for use of the platform, reinforce pain science education, and allow for personalized adjustment of the exercise component. They will also be used to monitor possible adverse events. The study has civil liability insurance.

3.2.2 Remote sessions

A total of 20 remote sessions will be delivered over 8 weeks, with 2 or 3 sessions per week. Each week, participants will be asked to: i) complete the task defined within the functional goal; ii) watch the educational videos and answer the corresponding quizzes (approximately 20 minutes); and iii) complete 3 exercise sessions on alternate days (approximately 30 minutes per session). The functional goal will focus on improving or recovering an activity of daily living that is considered important by the participant and limited by their pain. This component will be completed outside the InControl platform, using self-management strategies such as: i) pacing, which teaches participants to balance time spent in activity with time spent resting—for example, instead of preparing an entire meal at once, the participant works for five minutes and alternates this with five minutes of rest; or ii) gradual exposure to movement/activity, in which the participant begins the activity while standing for ten minutes and progressively increases the duration, for example by two additional minutes, according to increased tolerance. Overall, the goal is to facilitate self-management in activities that are meaningful to the participant. The educational content covers the definition of chronic pain, the role of the nervous system and sensitization, factors that modulate pain and contribute to its persistence (such as emotions and sleep), the distinction between pain and injury, and self-management strategies. This component, developed in a previous phase through co-creation with physicians, physical therapists, and older adults with pain, will be delivered through standardized videos, audio, and text. At the end of the videos, participants will answer the corresponding quizzes, consisting of questions about the content. Each participant will have an individualized exercise plan tailored to their abilities over the 8-week period. The plan will be repeated for two weeks, then reviewed and modified during the in-person sessions. Exercise intensity should be light (3–4) to moderate (5–6), measured using the Modified Borg Scale, which is used as a measure of perceived exertion[19,20]. Each exercise session will follow a standardized structure, lasting a total of 30 to 40 minutes, adjustable to each participant's tolerance and phase of the intervention. Sessions must include mobility and strengthening exercises and may also integrate other components. This structure should include a warm-up (5 minutes), a main component (15 to 30 minutes), and a cool-down (5 minutes).

3.3 Intervention fidelity assessment

Intervention fidelity refers to the extent to which an intervention is delivered as intended in the protocol[21,22] and will focus on the actions of the physical therapists delivering the InControl intervention. Two researchers will independently assess interventions recorded on the InControl platform, which will be randomly selected. The assessment will be based on a structured questionnaire, with responses rated on a Likert scale from 0 to 5, where 0 corresponds to "not at all confident" and 5 to "completely confident" regarding fidelity to the protocol.

3.4 Participant adherence to the intervention

Adherence to the InControl intervention is defined as the number of participants who complete the intervention[21,23], with completion of at least 75% of the sessions used as the adherence criterion. Adherence will be assessed through attendance records from the in-person sessions and electronic access data from the platform, including frequency and duration of use. If a participant misses two consecutive sessions, the research team will make a telephone contact to identify the reasons and provide the necessary support.

3.5 Intervention delivery

The intervention will be delivered by members of the research team and by physical therapists from the Local Health Unit of the Aveiro Region.

4. Assessment instruments and procedures

4.1 Primary variables

To assess the feasibility of the intervention, the following rates will be calculated: i) inclusion rate: the ratio between the number of participants included in the study and the total number of individuals contacted; ii) refusal rate: the ratio between the number of individuals who refused to participate in the study and the number of individuals contacted; iii) exclusion rate: the ratio between the number of individuals excluded for not meeting the inclusion criteria and the total number of individuals contacted; iv) dropout rate: the ratio between the number of participants who dropped out of the study and the number of participants who completed the baseline assessment; and v) retention rate: the ratio between the number of participants who completed the final assessment and the number of participants who completed the baseline assessment.

To assess usability, after the intervention (8 weeks), participants will be asked to complete the Portuguese version of the System Usability Scale regarding the InControl platform (Appendix – Interviews).

The acceptability and appropriateness of the procedures and intervention program will be assessed through a structured questionnaire and a semi-structured interview to explore participants' perceptions. The questionnaire will include 10 statements rated on a Likert scale from 1 to 5, where 1 corresponds to "strongly disagree" and 5 to "strongly agree." The interview guide will include questions about the appropriateness of the process, the structure of the program, the resources used, the experience with the InControl platform, and aspects related to integrating the program into the daily routine.

Only the audio from the interviews will be recorded. The recordings will be transferred to a University of Aveiro server and stored in an encrypted folder, where they will remain for a maximum of 2 weeks until transcription, after which they will be deleted (Appendix – Interviews).

InControl usage data, automatically recorded by the platform, such as the number of uses and duration of use, will be extracted. The time required to complete the assessment battery will also be estimated to determine its appropriateness.

4.2 Secondary variables

The Initiative on Methods, Measurement, and Pain Assessment in Clinical Trials (IMMPACT)[24] recommends the assessment of six domains in pain studies: i) pain—pain severity and interference, location and number of pain sites, and phenotype; ii) physical function—physical performance (handgrip strength, gait speed test, 5-times sit-to-stand test), and physical activity level; iii) emotional functioning—catastrophizing, kinesiophobia, and depressive symptoms; iv) perception of improvement—global perceived change, assessed post-intervention; v) symptoms and adverse

events—risk of sarcopenia and falls; and vi) detailed participant information—sociodemographic and clinical data, and quality of life. Evidence on the association between pain and cognitive impairment is conflicting in older adults with CMP[25,26]. Therefore, it is important to assess cognitive function both at baseline and post-intervention. The Portuguese versions of the instruments are valid and reliable (Appendix – Instruments).

5. Authentication and data management in InControl

Access to the InControl platform will be provided via a computer or tablet with internet access. Participants who do not have access to these resources will be provided with a tablet with internet access during the intervention period. Each participant will receive an access key to log in to the InControl platform, as well as instructions on how to use it safely at home. Initially, credentials will be assigned, and participants will be required to change their password at first login.

The principal investigator will create the access credentials for the physical therapists who will deliver the intervention, and each therapist will create user accounts for the participants they will treat, and only for those participants.

Data collected through InControl will relate only to system usage data and will be stored on a University of Aveiro server. The server complies with current regulations, regular backups will be performed, and the data will be encrypted.

Participant authentication in the system will be performed using a personal code. Only the researchers Anabela G. Silva and David Oliveira will have access to the key linking each participant to their corresponding identification code (pseudonymization). This code will be stored on researcher Anabela Silva's computer, which is owned by the University of Aveiro, in a password-protected Excel file.

6. Data analysis

Quantitative data will be analyzed using IBM SPSS Statistics version 31.0.0. Descriptive statistics will be used to characterize the sample, feasibility outcomes, and secondary variables. Means and standard deviations will be reported for continuous variables, and frequencies and percentages will be reported for ordinal and nominal variables.

Qualitative analysis will follow a three-step thematic analysis approach: reading, coding, and theme development[27]. Fully transcribed interviews will be independently analyzed by three researchers, whose summaries and concepts will then be compared[27]. During data coding, units of meaning (phrases) will be identified and categories will be grouped into common themes[27]. In addition, quantitative indicators will be provided regarding the number of participants who shared the ideas identified within each theme.

REFERENCES

1. Instituto Nacional de Estatística. Projeções de População Residente em Portugal. (2025). 2. Wettstein, M. & Tesarz, J. Increasing pain prevalence and intensity among middle-aged and older adults: Evidence from the German Ageing Survey. *Journal of Psychosomatic Research* **168**, 111233 (2023). 3. Domenichiello, A. F. & Ramsden, C. E. The silent epidemic of chronic pain in older adults. *Prog Neuropsychopharmacol Biol Psychiatry* **93**, 284–290 (2019). 4. Fong, H. P.-Y. *et al.* Determinants of health-related quality of life in older people with chronic musculoskeletal pain: a cross-sectional study. *BMC Geriatr* **24**, 119 (2024). 5. Cohen, S. P., Vase, L. & Hooten, W. M. Chronic pain: an update on burden, best practices, and new advances. *The Lancet* **397**, 2082–2097 (2021). 6. Silva, A. G., Santos, A. J., Andias, R. & Rocha, N. P. Effectiveness of digital pain management for older adults with musculoskeletal pain: systematic review with meta-analysis. *Front. Pain Res.* **6**, 1657014 (2025). 7. Valentijn, P. P. *et al.* Digital Health Interventions for Musculoskeletal Pain Conditions: Systematic Review and Meta-analysis of Randomized Controlled Trials. *J Med Internet Res* **24**, e37869 (2022). 8. Welsh, T. P., Yang, A. E. & Makris, U. E. Musculoskeletal Pain in Older Adults. *Med Clin North Am* **104**, 855–872 (2020). 9. Schofield, P. *et al.* Evidence-based clinical practice guidelines on the management of pain in older people – a summary report. *British Journal of Pain* **16**, 6–13 (2022). 10. Reid, M. C., Eccleston, C. & Pillemer, K. Management of chronic pain in older adults. *BMJ* https://doi.org/10.1136/bmj.h532 (2015) doi:10.1136/bmj.h532. 11. Horgas, A. L. Pain Management in Older Adults. *Nursing Clinics of North America* https://doi.org/10.1016/j.cnur.2017.08.001 (2017) doi:10.1016/j.cnur.2017.08.001. 12. Kechichian, A. *et al.* Multimodal Interventions Including Rehabilitation Exercise for Older Adults With Chronic Musculoskeletal Pain: A Systematic Review and Meta-analyses of Randomized Controlled Trials. *Journal of Geriatric Physical Therapy* **45**, 34 (2022). 13. Silva, H. de J. *et al.* Group-based exercise reduces pain and disability and improves other outcomes in older people with chronic non-specific low back pain: the ESCAPE randomised trial. *Journal of Physiotherapy* **71**, 108–116 (2025). 14. Syroyid Syroyid, I., Cavero-Redondo, I. & Syroyid Syroyid, B. Effects of Resistance Training on Pain Control and Physical Function in Older Adults With Low Back Pain: A Systematic Review With Meta-analysis. *Journal of Geriatric Physical Therapy* **46**, E113 (2023). 15. Heleno, E., Andias, R. & Silva, A. G. What do community-dwelling older adults with chronic

pain value in a program of combined pain neuroscience education plus exercise? *Patient Education and Counseling* **104**, 3072–3078 (2021). 16. Siddall, B. *et al.* Short-term impact of combining pain neuroscience education with exercise for chronic musculoskeletal pain: a systematic review and meta-analysis. *PAIN* **163**, e20 (2022). 17. Eldridge, S. M. *et al.* CONSORT 2010 statement: extension to randomised pilot and feasibility trials. *BMJ* i5239 (2016) doi:10.1136/bmj.i5239. 18. Perrot, S. *et al.* The IASP classification of chronic pain for ICD-11: chronic secondary musculoskeletal pain. *PAIN* **160**, 77 (2019). 19. Riebe, D. *et al.* Updating ACSM's Recommendations for Exercise Preparticipation Health Screening. *Medicine & Science in Sports & Exercise* **47**, 2473 (2015). 20. Baptista, F. M., Andias, R., Rocha, N. P. & Silva, A. G. A Practice Guide for Physical Therapists Prescribing Physical Exercise for Older Adults. *Journal of Aging and Physical Activity* **32**, 771–783 (2024). 21. Páez, A., Nunan, D., McCulloch, P. & Beard, D. The influence of intervention fidelity on treatment effect estimates in clinical trials of complex interventions: a metaepidemiological study. *Journal of Clinical Epidemiology* **177**, 111610 (2025). 22. Teresi, J. A., Yu, X., Stewart, A. L. & Hays, R. D. Guidelines for Designing and Evaluating Feasibility Pilot Studies. *Med Care* **60**, 95–103 (2022). 23. Chan, C. L., Taljaard, M., Lancaster, G. A., Brehaut, J. C. & Eldridge, S. M. Pilot and feasibility studies for pragmatic trials have unique considerations and areas of uncertainty. *J Clin Epidemiol* **138**, 102–114 (2021). 24. Dworkin, R. H. *et al.* Core outcome measures for chronic pain clinical trials: IMMPACT recommendations. *Pain* **113**, 9–19 (2005). 25. Yuan, H. *et al.* Contribution of pain to subsequent cognitive decline or dementia: A systematic review and meta-analysis of cohort studies. *International Journal of Nursing Studies* **138**, 104409 (2023). 26. de Aguiar, G. P. C. G. *et al.* Persistent pain and cognitive decline in older adults: a systematic review and meta-analysis from longitudinal studies. *PAIN* **161**, 2236 (2020). 27. Saunders, C. H. *et al.* Practical thematic analysis: a guide for multidisciplinary health services research teams engaging in qualitative analysis. *BMJ* **381**, e074256 (2023).
