# Supplementary material for: Feasibility, acceptability and usability of InControl web-platform intervention for older adults with chronic musculoskeletal pain: A mixed-methods pilot study protocol
Source: PLoS One. 2026 Oct 14;21(10):e0360321. doi: 10.1371/journal.pone.0360321 (PMC13667814; doi:10.1371/journal.pone.0360321)
Supplement: Supplementary file 4 [file pone.0360321.s004.pdf]

QUESTIONÁRIO DA EXPERIÊNCIA DOS PARTICIPANTES

Gostaríamos de saber a sua opinião sobre a intervenção InControl. A sua perspetiva é fundamental para compreendermos a aceitabilidade da intervenção.

Para cada uma das afirmações abaixo, por favor, assinale a sua resposta de 1 a 5, de acordo com a seguinte escala:

1 – Discordo totalmente

2 – Discordo

3 – Não concordo nem discordo

4 – Concordo

5 – Concordo totalmente

	Discordo totalmente	Discordo	Não discordo nem concordo	Concordo	Concordo totalmente
1. A combinação de sessões presenciais e à distância é adequada às minhas capacidades e rotinas	1	2	3	4	5
2. A linguagem, os exemplos e as metáforas utilizadas foram fáceis de compreender	1	2	3	4	5
3. Tenho total confiança na informação e estratégias dadas pelo fisioterapeuta para gestão da dor	1	2	3	4	5
4. Foi fácil gerir o tempo necessário para a intervenção de modo a gerir a minha rotina	1	2	3	4	5
5. Considero que o esforço e tempo dedicados a esta intervenção valeram a pena	1	2	3	4	5
6. Recomendaria a intervenção InControl a outras pessoas com dor crónica	1	2	3	4	5
7. Sinto que a intervenção InControl foi relevante para mim	1	2	3	4	5
8. A intervenção InControl ajudou-me a aumentar o conhecimento e compreensão sobre a minha dor	1	2	3	4	5
9. Os conteúdos da intervenção mudaram positivamente a forma como eu penso sobre a dor e o movimento	1	2	3	4	5
10. Sinto-me motivado e capaz de manter as estratégias e exercícios a longo prazo	1	2	3	4	5
