# Supplementary material for: Feasibility, acceptability and usability of InControl web-platform intervention for older adults with chronic musculoskeletal pain: A mixed-methods pilot study protocol
Source: PLoS One. 2026 Oct 14;21(10):e0360321. doi: 10.1371/journal.pone.0360321 (PMC13667814; doi:10.1371/journal.pone.0360321)
Supplement: Supplementary file 5 [file pone.0360321.s005.pdf]

PARTICIPANT EXPERIENCE QUESTIONNAIRE

We would like to know your opinion about the InControl intervention. Your perspective is essential to help us understand the acceptability of the intervention.

For each of the statements below, please indicate your response from 1 to 5, according to the following scale:

1 – Strongly disagree

2 – Disagree

3 – Neither agree nor disagree

4 – Agree

5 – Strongly agree

	**Discordo totalmente**	**Discordo**	**Não discordo nem concordo**	**Concordo**	**Concordo totalmente**
**1.** The combination of in-person and remote sessions is appropriate for my abilities and routines.	1	2	3	4	5
**2.** The language, examples, and metaphors used were easy to understand.	1	2	3	4	5
**3.** I have complete confidence in the information and pain-management strategies provided by the physical therapist.	1	2	3	4	5
**4.** It was easy to manage the time required for the intervention while maintaining my usual routine.	1	2	3	4	5
**5.** I believe that the effort and time I devoted to this intervention were worthwhile.	1	2	3	4	5
**6.** I would recommend the InControl intervention to other people with chronic pain.	1	2	3	4	5
**7.** I feel that the InControl intervention was relevant to me.	1	2	3	4	5
**8.** The InControl intervention helped me increase my knowledge and understanding of my pain.	1	2	3	4	5
**9.** The content of the intervention positively changed the way I think about pain and movement.	1	2	3	4	5
**10.** I feel motivated and capable of maintaining the strategies and exercises over the long term.	1	2	3	4	5
