# Supplementary material for: Feasibility, acceptability and usability of InControl web-platform intervention for older adults with chronic musculoskeletal pain: A mixed-methods pilot study protocol
Source: PLoS One. 2026 Oct 14;21(10):e0360321. doi: 10.1371/journal.pone.0360321 (PMC13667814; doi:10.1371/journal.pone.0360321)
Supplement: Supplementary file 6 [file pone.0360321.s006.pdf]

GUIÃO DAS ENTREVISTAS

As entrevistas serão realizadas de forma individual, num ambiente calmo.

Introdução

Agradecimento pela disponibilidade

Explicar o objetivo da entrevista. A sua resposta será importante para entender e melhorar a experiência na utilização do InControl; as respostas não são certas ou erradas.

Reafirmar o anonimato e pedir autorização para gravar a entrevista.

Questões a fazer ao participante

O que pensa das informações recebidas antes do estudo? O que pensa do tempo de espera entre o contato e o início da intervenção?

O que achou mais útil ou positivo?

O que achou menos útil ou mais difícil? (quais foram as principais barreiras?)

Qual foi o maior desafio para tentar manter a rotina do programa no seu dia a dia?

Como descreveria a navegação na plataforma?

Tem alguma sugestão sobre como o formato das sessões de intervenção poderia ser melhorado? (por exemplo: número de sessões, duração das sessões)

Como descreveria a sua experiência geral com o programa/intervenção?

Encerramento

Há algo mais da sua experiência que gostasse de partilhar, que ainda não tenhamos abordado?

Explicar que iremos transcrever e analisar os dados. Pedir se, após a análise, podemos encaminhar os dados para que possam confirmar as ideias abordadas.

Agradecer novamente a disponibilidade e participação no estudo.
