# Supplementary material for: Feasibility, acceptability and usability of InControl web-platform intervention for older adults with chronic musculoskeletal pain: A mixed-methods pilot study protocol
Source: PLoS One. 2026 Oct 14;21(10):e0360321. doi: 10.1371/journal.pone.0360321 (PMC13667814; doi:10.1371/journal.pone.0360321)
Supplement: Supplementary file 7 [file pone.0360321.s007.pdf]

SEMI-STRUCTURED INTERVIEW GUIDE

The interviews will be conducted individually in a quiet and comfortable setting.

Introduction

Thank the participant for their availability and willingness to participate.

Explain the purpose of the interview. Explain that their responses will be important for understanding and improving the experience of using InControl, and that there are no right or wrong answers.

Reaffirm the confidentiality and anonymity of the participant's responses and ask for permission to audio-record the interview.

Questions for the Participant

What did you think about the information you received before the study?

What did you think about the waiting time between the initial contact and the start of the intervention?

What did you find most useful or positive about the intervention?

What did you find least useful or most difficult? What were the main barriers you experienced?

What was the biggest challenge you faced in trying to maintain the program routine in your daily life?

How would you describe your experience navigating the platform?

Do you have any suggestions for how the format of the intervention sessions could be improved? *For example, the number or duration of sessions.*

How would you describe your overall experience with the program/intervention?

Closing

Is there anything else about your experience that you would like to share that we have not yet discussed?

Explain that the interviews will be transcribed and the data will be analyzed. Ask the participant whether they would be willing, after the analysis, to review the findings and confirm whether they accurately reflect the ideas and experiences they discussed.

Thank the participant again for their time and participation in the study.
